# Pembrolizumab-induced myasthenia gravis-like disorder, ocular myositis, and hepatitis: a case report

**DOI:** 10.1186/s13256-021-02722-8

**Published:** 2021-04-30

**Authors:** Chia-Yi Tian, Yang-Hao Ou, Shih-Liang Chang, Chih-Ming Lin

**Affiliations:** 1 Department of Neurology, Changhua Christian Hospital, No. 135, Nanxiao Street, Changhua, 500 Taiwan; 2 Department of Social Work and Child Welfare, Providence University, Taichung, Taiwan; 3 Department of Medicinal Botanicals and Health Applications, Da-Yeh University, No.168, University Road, Changhua, Taiwan; 4 School of Chinese Medicine, China Medical University, Taichung, Taiwan

**Keywords:** Pembrolizumab, Immune checkpoint inhibitor, PD-1, Neuroinflammation, Ocular myositis, Hepatitis, Case report

## Abstract

**Introduction:**

Pembrolizumab and other immune checkpoint inhibitors are the emerging treatment for selected, high-grade malignancies. However, a small number of patients are unable to tolerate its adverse effects, leading to discontinuation of this potentially life-changing therapy. In this study, we present a case of high-grade urothelial carcinoma patient, who experienced neurocomplications during the first pembrolizumab administration. However, we were able to limit the adverse effect by concomitant use of low-dose oral steroids.

**Case presentation:**

A 75-year-old Taiwanese female with high-grade urothelial carcinoma of the left ureter came to the neurology clinic with complaints of acute onset of bilateral ptosis 16 days after her first infusion of pembrolizumab. It was found that she developed complete bilateral ptosis and limited extraocular muscle movements. Myasthenia gravis-related antibodies and repetitive stimulation test were negative. We diagnosed her with pembrolizumab-induced myasthenia gravis-like disorder and myositis based on clinical symptoms and elevation of muscle enzymes. We commenced methylprednisolone pulse therapy followed by oral steroid therapy with gradual resolution of the symptoms. Three months later, the patient received a second cycle of pembrolizumab with low-dose oral steroids without any complications.

**Conclusion:**

Pembrolizumab exerts its antitumor activity by interfering with the binding of programmed death 1 and its ligand, programmed death ligand 1. As a result, enhanced cytotoxic T cells can recognize tumor cells and induce cellular death. However, neurological complications may be severe and require prompt recognition and treatment. Our case demonstrated that concomitant use of low-dose steroids and pembrolizumab might prevent such complications.

## Introduction

Immune checkpoint inhibitors (ICI) such as cytotoxic T-lymphocyte antigen 4 (CTLA-4), programmed cell death 1 (PD-1), and programmed cell death ligand 1 (PD-L1) are increasingly prevalent in the treatment of a plethora of cancer types. At the same time, ICI-related adverse effects have caught many people's attention. The adverse effects can involve any system of the body [1], even though only 3% of patients experienced neurological complications, but the sequelae can be permanent [2]. The management of severe neurocomplications often requires pembrolizumab discontinuation and initiation of immunosuppressants. We reported a case of myasthenia gravis-like disorder, ocular myositis, and hepatitis after the infusion of the first pembrolizumab dose, and discuss the potential role of low-dose oral steroids given concomitantly with pembrolizumab to prevent neurological complications in the subsequent cycle.

## Case presentation

**Fig. 1:**
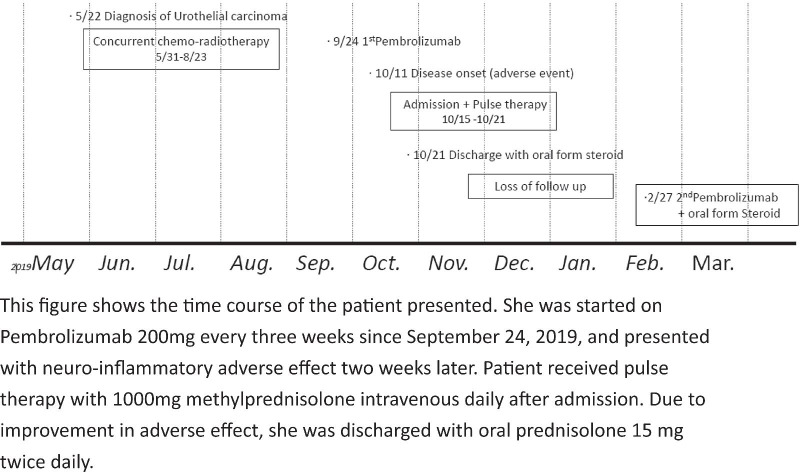
This figure shows the time course of the patient presented. She was started on pembrolizumab 200 mg every three weeks from September 24, 2019, and presented with neuroinflammatory adverse effect 2 weeks later. The patient received pulse therapy with 1000 mg methylprednisolone intravenous daily after admission. Because of improvement in adverse effect, she was discharged with oral prednisolone 15 mg twice daily.

A 75-year-old Taiwanese female presented with painless hematuria and unilateral left leg edema. Her underlying conditions include type 2 diabetes mellitus, hyperlipidemia, and hypertension. Initial assessment by the urologist diagnosed her with urothelial carcinoma of the left ureter, for which she underwent left nephroureterectomy. However, the surgeon discovered partial nonresectable tumors on the pelvic wall and upgraded her tumor staging to pT4N2. She received five cycles of concurrent chemoradiotherapy (6000 cGy/30 fx) with cisplatin and gemcitabine after the surgery. Because she responded poorly to cisplatin-based therapy and the tumor cells tested positive for PD-L1 (>5%), she was started on pembrolizumab (200 mg) once every three weeks (Fig. 1).

She presented to the neurology clinic with an acute onset of bilateral ptosis 16 days after her first infusion of pembrolizumab. Neurological examination demonstrated that she developed complete bilateral ptosis (right ptosis developed one day before left ptosis) without noticeable diurnal change. Extraocular muscle movement (EOM) showed all-direction limitation with vertical and mild adduction sparing. Symmetric pupil reflex was reactive, and no diplopia, orbital pain, chemosis of the conjunctiva, proptosis, facial numbness, muscle pain, or dyspnea were noted. Additionally, there was no bulbar, extremity, or axial involvement.

**Fig. 2:**
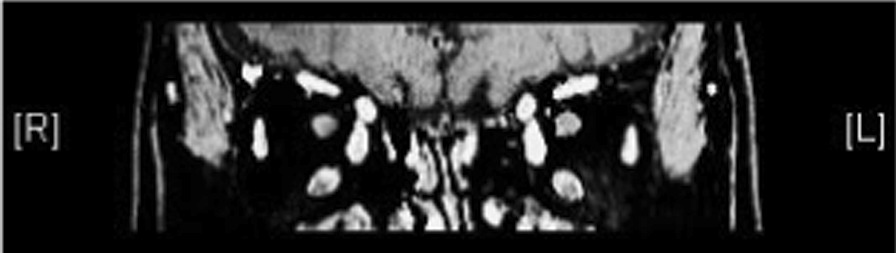
Gadolinium-enhanced T1-weighted MRI image of bilateral orbicularis oculi muscle, coronal view. Showing no abnormal mass lesions or enhancement

**Fig. 3:**
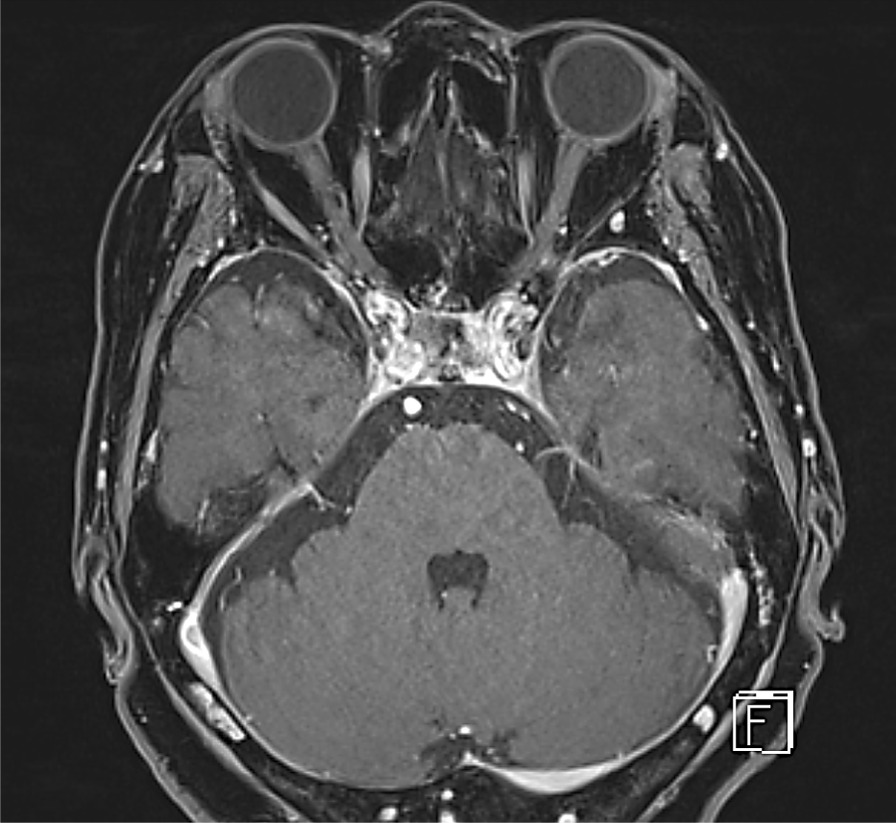
Gadolinium-enhanced T1-weighted MRI image showing no abnormal mass lesions or enhancement within the cavernous sinus, axial view

Laboratory data showed elevated liver enzymes (alanine aminotransferase 163 IU/L, aspartate aminotransferase 258 IU/L) and creatine phosphokinase level (4817 IU/L). Her liver enzymes were within normal range one week before the onset of symptoms. Erythrocyte sedimentation rate and thyroid function were normal. Autoimmune antibodies were negative for the anti-acetylcholine receptor, anti-striated muscle, and anti-muscle-specific kinase antibodies. Gadolinium-enhanced magnetic resonance imaging (MRI) found no evidence of inflammatory, infection, or mass lesions, especially in her orbital region (Figs. 2, 3). We also performed a cerebrospinal fluid analysis to rule out neuromuscular junction disorders, paraneoplastic disorders, and peripheral neuropathies. Repetitive stimulation test (3 Hz, abductor digiti minimi (ADM), trapezius, unable for eye because of lack of technical capabilities), ice pack test, and computed tomography of the chest and mediastinum were unremarkable.

After admission, she was treated with pyridostigmine 120 mg daily because of suspected myasthenia gravis; however, it was ineffective. Thus, we commenced methylprednisolone pulse therapy (1000 mg intravenous daily for 4 days) for pembrolizumab-induced neuroinflammatory symptoms (CTCAE grade III). The patient's ptosis and EOM improved gradually, and her liver enzymes returned to baseline upon follow-up (CTCAE grade I). She was then discharged with oral prednisolone 15 mg twice daily. Because of this event, her urologist held the pembrolizumab treatment, and she was subsequently lost to follow-up.

**Fig. 4:**
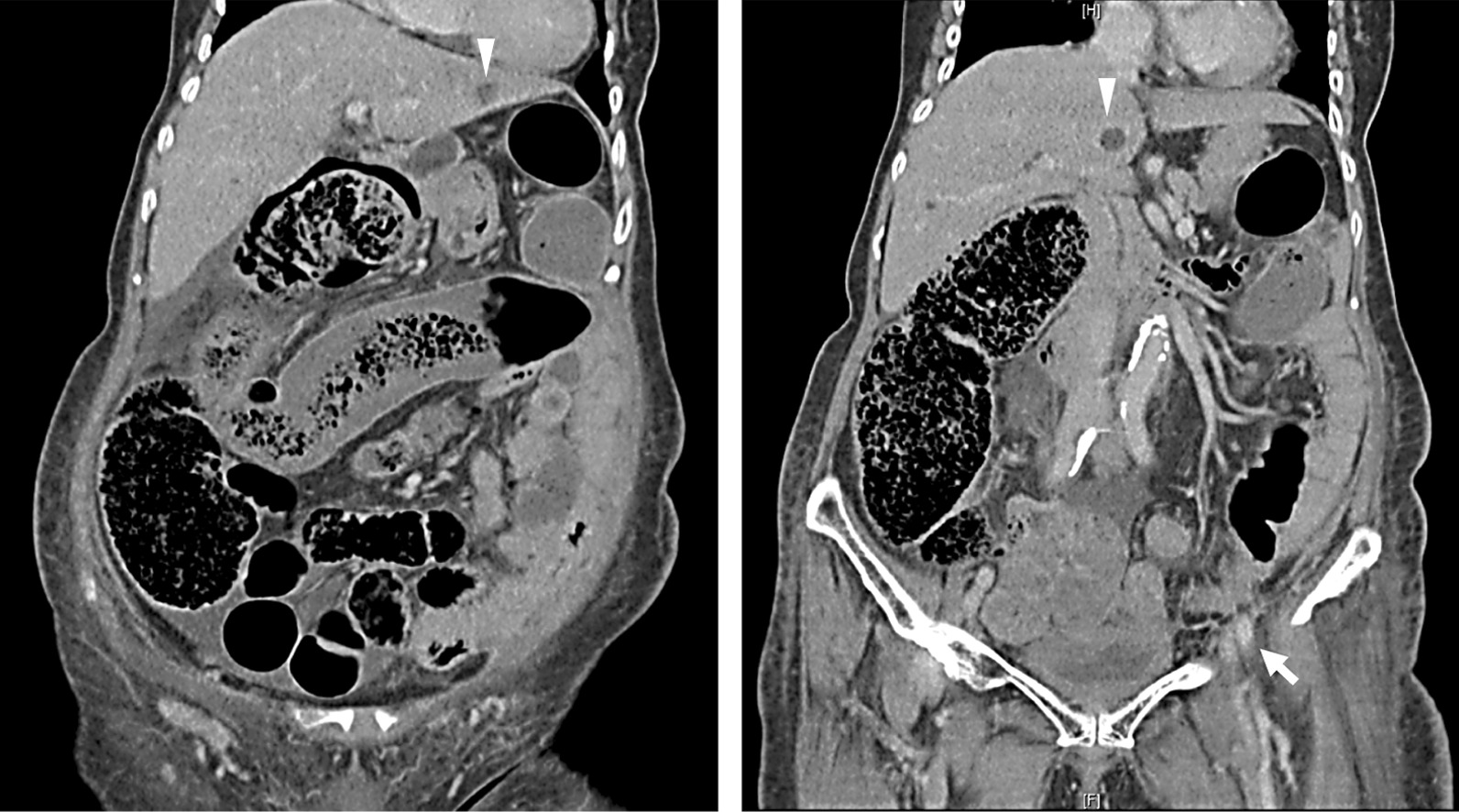
CT scan of the abdomen with/without contrast, showing distended colon with obstruction at the sigmoid colon. Increased infiltrative soft tissues near the left external iliac vessels favoring tumor metastasis (white arrow). Solid nodule in the liver (white arrow head) also suggestive of metastasis

Three months later, the patient was readmitted to the oncology ward for progression of urothelial cancer, showing lung, liver, and abdomen metastasis with severe obstructive ileus (Fig. 4). After careful consideration, pembrolizumab was restarted as a last resort with the continuation of oral prednisolone 15 mg twice daily. Two weeks after the second infusion of pembrolizumab, no apparent signs of ptosis, EOM limitation, or other neuroinflammatory symptoms were found.

## Discussion and conclusion

Pembrolizumab is an immune checkpoint inhibitor (ICI) that has antitumor activity in high-grade urothelial cancer [3]. Physiologically, all activated T cells carry programmed death 1 (PD-1) protein on their surfaces. The binding of PD-1 with its ligand, PD-L1, leads to immune tolerance to contain the level of local inflammation and avoid collateral damage to healthy tissues [4]. A study has shown that locally aggressive urothelial cancer overexpresses PD-L1, leading to the downregulation of cytotoxic T cells and escape from immune surveillance [5]. Pembrolizumab is an anti-PD-1 drug that interferes with the binding of PD-L1 expressed by the cancer cells to the PD-1 on the cytotoxic T cells, allowing T-cell-mediated cell killing [6].

The side effects of pembrolizumab therapy vary widely, and multiple systems can be involved, including musculoskeletal, cardiac, pulmonary, and nervous systems [7]. Pembrolizumab's ability to upregulate immune response may have contributed to these adverse events. Several possible mechanisms were proposed in a review article by Postow *et al.*, such as enhanced T-cell activity toward common antigens that are expressed in both healthy and tumor cells, or exaggerated immune response through increased production of cytokines and complement-mediated pathway [8]. A study showed that 3% of the patients who received anti-PD-1 drugs (pembrolizumab or nivolumab) experienced neurological complications [2], with neuromuscular disorders being the most frequent complaints, ranging from myasthenia gravis (MG) and Guillain–Barre syndrome to autoimmune myopathies [9]. Treatment of neurological adverse effects generally requires the discontinuation of ICI and prompt administration of high-dose corticosteroids, plasmapheresis, or intravenous immunoglobulin (IVIG) [10].

Kamo *et al.* described two cases with pembrolizumab-related systemic myositis including ptosis and limited EOM, both receiving intravenous methylprednisolone with subsequent amelioration of the symptoms [11]. In another case study, the patient was diagnosed with severe ICI-related myositis; despite aggressive treatment of IVIG, the patient died from cardiac involvement. [12]. However, these studies did not mention the ensuing management of cancer nor suggest to substitute pembrolizumab therapy with an alternative.

Similar to the cases mentioned above, our patient was treated with methylprednisolone therapy with an improvement of the symptoms; additionally, she received low-dose (15 mg) oral prednisolone maintenance therapy. Later, our patient was started on a second infusion of pembrolizumab out of desperation and in the hope to ameliorate the symptoms caused by the wide spread of urothelial cancer. We observed no clinically significant symptoms suggesting pembrolizumab-related complications at 2 weeks follow-up.

Some experts have suggested that the therapeutic effect of immunotherapy may be counteracted by corticosteroids [13]. However, a recent systematic review, comprising 27 articles and a total of 72 patients who were reported to be on pembrolizumab and steroids, concluded that no objective data suggest that concomitant use of corticosteroids leads to a poorer clinical outcome [14]. It is suspected that there are additional mechanisms or biomolecular pathways yet to be elucidated that contribute to ICI's therapeutic effect [15]. While there is no clear evidence indicating that low-dose maintenance steroids can prevent ICI-induced myositis, this case demonstrates that, after careful evaluation and informed consent, first-line physicians may attempt treatment of advanced-stage urothelial cancer concomitantly with pembrolizumab and low-dose steroids in patients for whom neuroinflammatory side effects may be an issue.

## Data Availability

Data sharing is not applicable to this article as no datasets were generated or analyzed during the current study.

## Abbreviations

AMD: Abductor digiti minimi
CTCAE: Common terminology criteria for adverse events
CTLA-4: Cytotoxic T-lymphocyte antigen 4
EOM: Extraocular muscle movement
ICI: Immune checkpoint inhibitors
IVIG: Intravenous immunoglobulin
MG: Myasthenia gravis
MRI: Magnetic resonance imaging
PD-1: Programmed death 1
PD-L1: Programmed death ligand 1
